# Pycnodysostosis: Clinical Insights From Two Siblings

**DOI:** 10.7759/cureus.69609

**Published:** 2024-09-17

**Authors:** Aziza Elouali, Hajar Elmoqaddem, Massilia Bouhmidi, Maria Rkain, Abdeladim Babakhouya

**Affiliations:** 1 Department of Pediatrics, Faculty of Medicine and Pharmacy, Mohammed I University, Oujda, MAR; 2 Department of Pediatrics, Mohammed VI University Hospital, Oujda, MAR; 3 Department of Pediatric Gastroenterology, Mohammed VI University Hospital, Oujda, MAR

**Keywords:** osteosclerosis, pycnodysostosis, rare, short stature, syndrome

## Abstract

Pycnodysostosis is a rare autosomal recessive bone disorder caused by mutations in the cathepsin K (CTSK) gene, characterized by increased bone density, short stature, and skeletal fragility. This study reports on two siblings from a consanguineous marriage, observed at the Mohammed VI University Hospital in Oujda, Morocco. Both patients presented with typical symptoms, including craniofacial dysmorphism and skeletal abnormalities. Radiographic findings confirmed increased bone density and acro-osteolysis. The cases highlight the importance of early and accurate diagnosis, comprehensive management to address the broad spectrum of clinical manifestations, and genetic counseling to inform family planning and manage the risk of recurrence in familial pycnodysostosis.

## Introduction

Pycnodysostosis is a rare sclerosing bone disorder first described in 1962 [1]. This hereditary condition is caused by mutations in the CTSK gene, which encodes cathepsin K, an enzyme essential for bone resorption [2]. These genetic mutations result in a variety of clinical manifestations, including growth retardation, skeletal abnormalities, and recurrent fractures. Patients typically exhibit short stature, cranial dysplasia, and orofacial abnormalities, contributing to a heterogeneous clinical presentation [3].

We report two sisters with pycnodysostosis, observed in the pediatric department of the Mohammed VI University Hospital in Oujda, Morocco. Both presented with clinical and radiographic features characteristic of this rare disorder.

## Case presentation

Case 1

**Figure 1:**
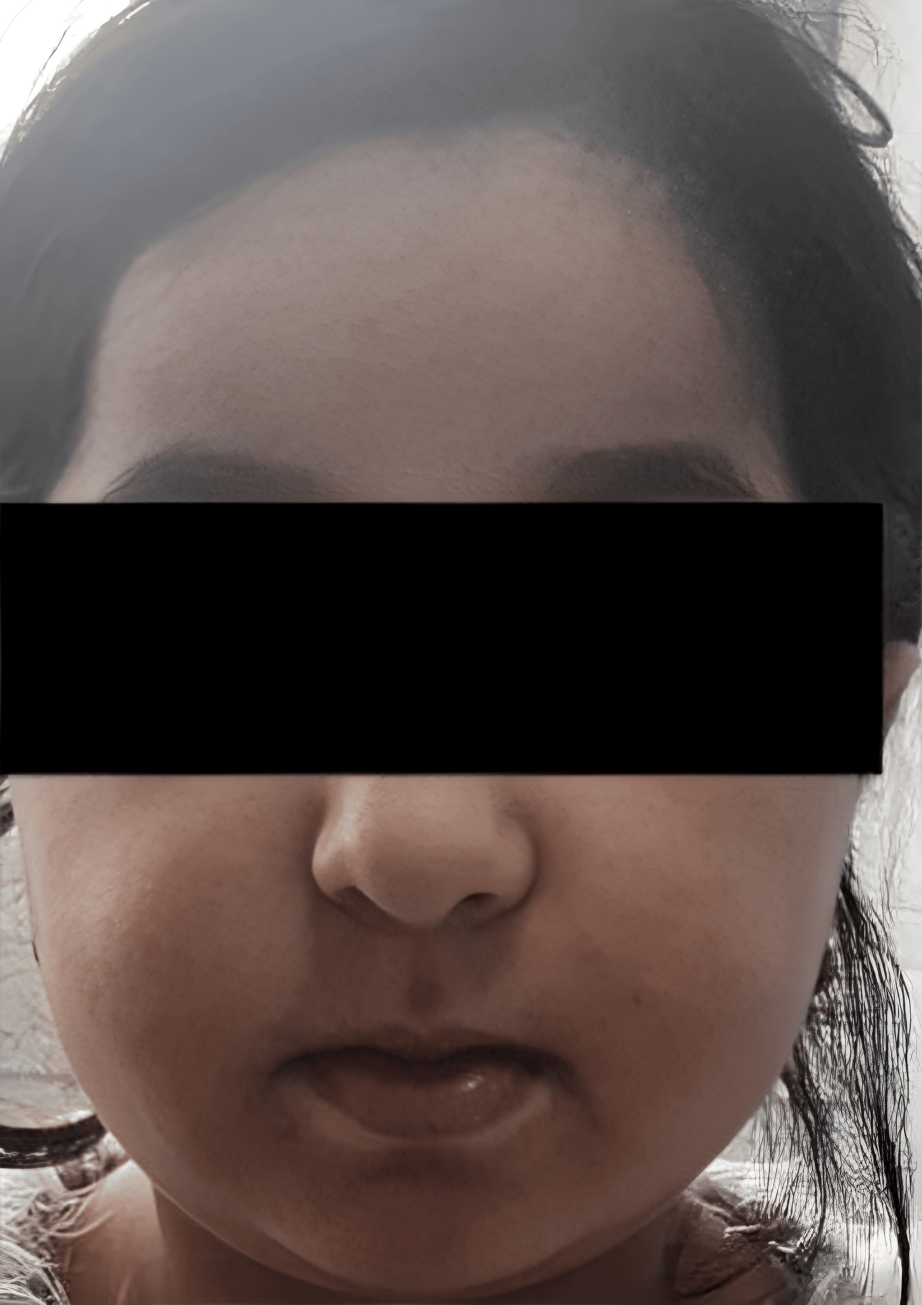
Patient's facial features Small, narrow face, prominent cheeks, beaked nose, deep nasolabial skin folds, and micrognathia.

**Figure 2:**
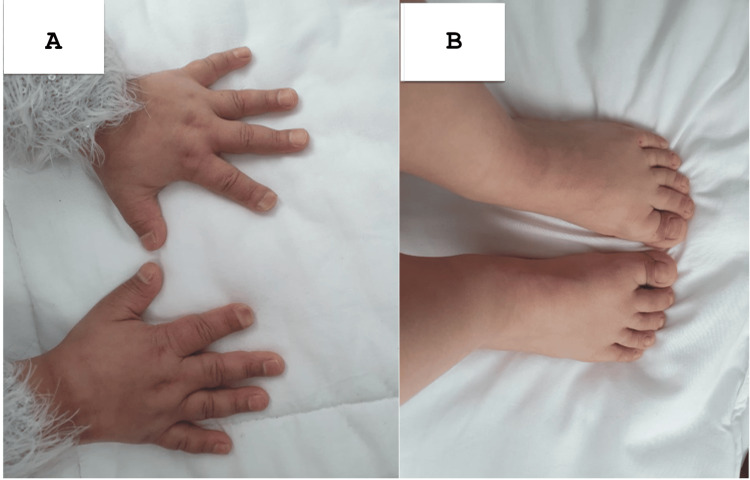
Patient's hands (A) and feet (B) Short terminal phalanges with koilonychia.

**Figure 3:**
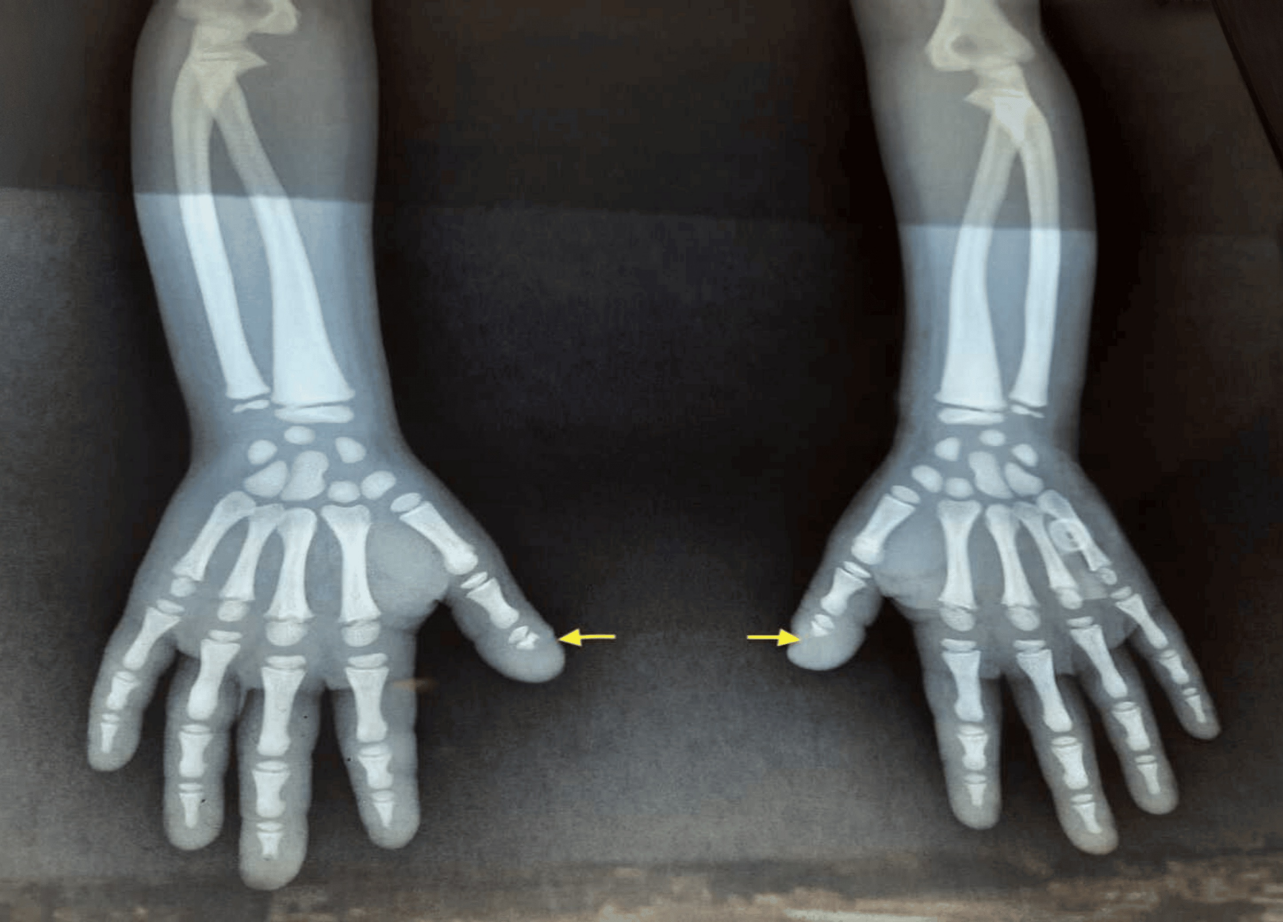
Radiograph of the both hands showing acro-osteolysis (arrow)

**Figure 4:**
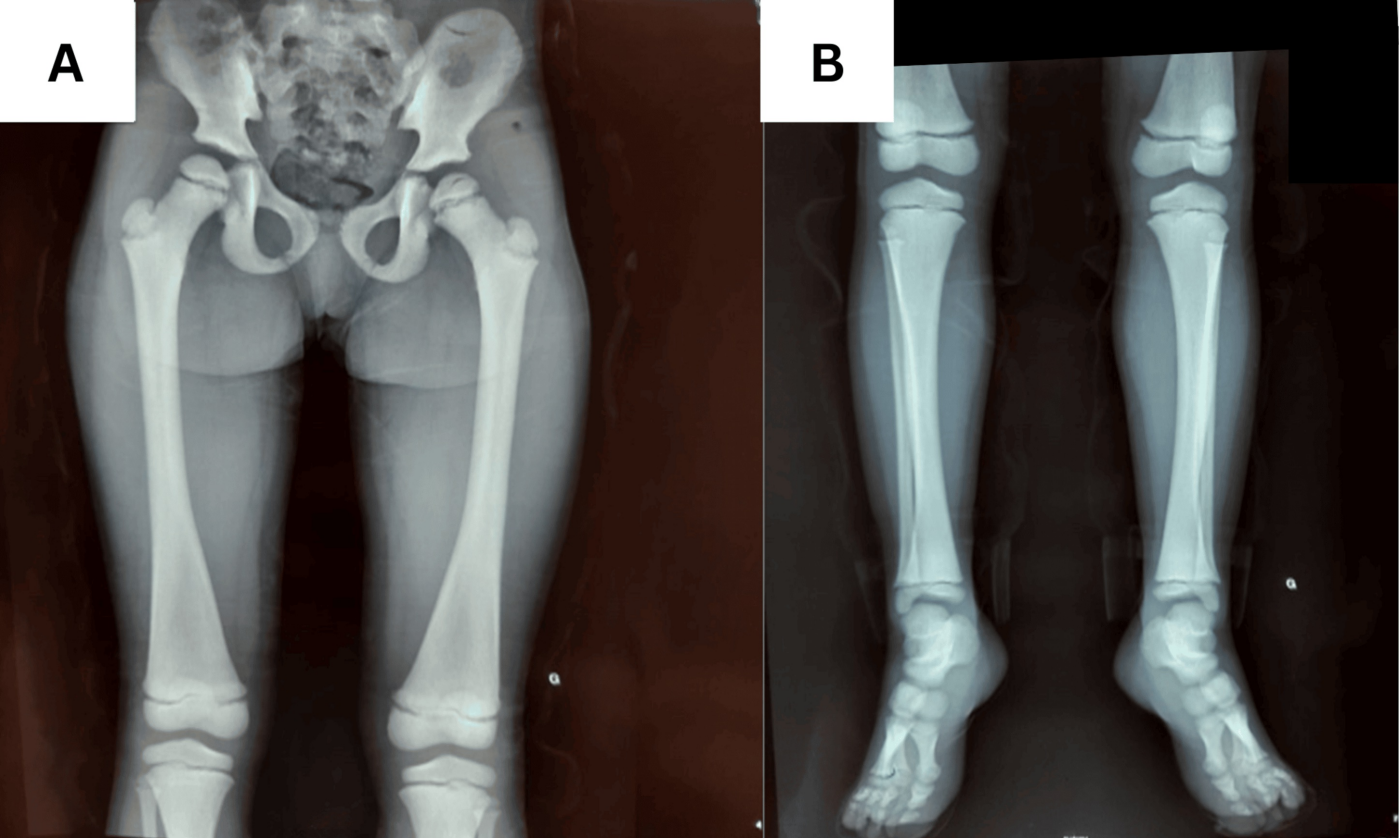
Radiograph of the pelvis and femurs (right and left) (A) and legs and feet (right and left) (B) in anteroposterior view showing increased bone density

A 12-year-old girl, born to first-degree consanguineous parents, was admitted for evaluation of growth retardation. She was attending school with normal academic performance. The patient had a history of repeated fractures at the mid-shaft of the right tibia, each following minor trauma. Clinical examination revealed severe growth retardation, with a height of 118 cm, accompanied by dysmorphic features, including a small face, broad forehead, frontal bossing (Figure 1), and a persistent anterior fontanelle. Oral examination showed a high-arched palate and dental crowding. Her hands and feet appeared coarse and short (Figure 2). Systemic examination revealed no organomegaly. Radiographs of both hands showed lysis of the distal phalanges of the fingers (Figure 3) and increased bone density (Figure 4). The patient's bone age was estimated to be 8.

Case 2

**Figure 5:**
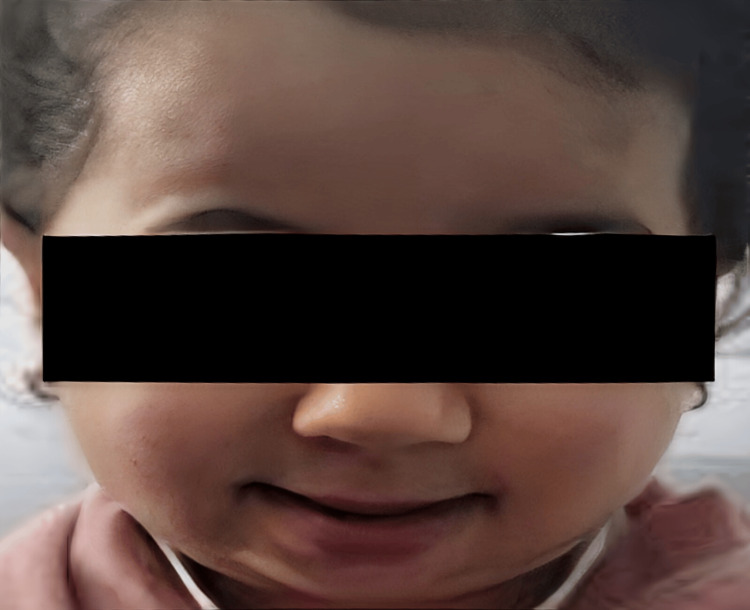
Facial features of the younger sibling (case 2) showing similar characteristics Frontal bossing, prominent cheeks, beaked nose, and micrognathia.

The younger sister of the first patient, aged six years, presented with growth retardation below four standard deviations and exhibited a similar clinical profile. She had a height of 92 cm, facial dysmorphism including a broad forehead, frontal bossing, a persistent anterior fontanelle, and micrognathia (Figure 5). Additional findings included finger malformations, dental misalignment with multiple caries, curved nails, and a scoliotic posture of the dorsal spine. Radiographic analysis also revealed increased bone density.

The clinical manifestations and radiological findings in both sisters were suggestive of pycnodysostosis. Both were enrolled in a long-term follow-up program, including orthopedic care to minimize fracture risk, dental hygiene, and periodic dental evaluations to manage caries.

## Discussion

Pycnodysostosis is a rare hereditary disorder with an estimated prevalence of one to three cases per million individuals [3]. Approximately 20% of cases occur in children born from consanguineous marriages, reflecting the autosomal recessive inheritance pattern of the disease [2]. This condition arises from mutations in the CTSK gene on chromosome 1q21, which encodes cathepsin K, a lysosomal protease crucial for bone resorption. These mutations impair the degradation of type 1 collagen, a major component of the organic bone matrix, leading to increased bone density and frequent fractures from minor trauma [2]. In some cases, frequent fractures can be the first indication of the disease [4], as observed in our first patient.

Diagnosing pycnodysostosis primarily relies on clinical presentation and radiographic findings [5]. Clinically, the disorder is marked by distinctive craniofacial dysmorphism and orofacial features, including frontal, occipital, and parietal bossing; low-set ears; prominent cheeks; a high nasal bridge; a beaked nose; and hypoplasia of the maxilla and mandible. Additional manifestations include dental anomalies, short stature, broad hands and feet with dystrophic nails, and trunk deformities such as scoliosis and increased lumbar lordosis [5,6].

Radiographic evaluation typically reveals generalized skeletal sclerosis. Other findings may include an open anterior fontanelle, unclosed sutures, small facial bones, non-pneumatized paranasal sinuses, and a flattened mandibular angle. Terminal phalanges of the hands may be partially or completely aplastic, with loss of ungual tufts [5,7].

Accurate differentiation from other conditions such as osteopetrosis, cleidocranial dysplasia, and idiopathic acroosteolysis is essential for proper management [5]. The combination of acroosteolysis and osteosclerosis suggests pycnodysostosis [5].

Management of pycnodysostosis requires a multidisciplinary approach. As there is no specific treatment available, symptomatic management strategies are recommended [6]. These strategies include fracture prevention, maintaining good oral hygiene, and providing psychological support to ensure optimal growth and development. Although genetic counseling has been suggested for the family, mutation testing remains unavailable in our current context.

## Conclusions

Pycnodysostosis presents a range of clinical challenges that necessitate a tailored management approach. Early and accurate diagnosis is critical to mitigate complications and ensure effective monitoring. The familial nature of pycnodysostosis, particularly in consanguineous marriages, underscores the significant role of genetic counseling in guiding family planning and assessing the risk of recurrence. This study highlights the need for ongoing research to further understand the condition and improve patient outcomes.
